# A Practical Review of the Emerging Direct Anticoagulants, Laboratory Monitoring, and Reversal Agents

**DOI:** 10.3390/jcm7020029

**Published:** 2018-02-11

**Authors:** Stephanie A. Joppa, Justin Salciccioli, Jill Adamski, Salma Patel, Waldemar Wysokinski, Robert McBane, Farah Al-Saffar, Heidi Esser, Fadi Shamoun

**Affiliations:** 1 Department of Cardiology, Mayo Clinic, Phoenix, AZ 85054, USA; joppa@umn.edu (S.A.J.); al-saffar.farah@mayo.edu (F.A.-S.); 2 Whipps Cross University Hospital, London E11 1NR, UK; justin.salciccioli12@imperial.ac.uk; 3 Department of Laboratory Medicine and Pathology, Mayo Clinic, Phoenix, AZ 85054, USA; adamski.jill@mayo.edu; 4 Center for Sleep Medicine, Mayo Clinic, Rochester, MN 55905, USA; salmapatelaz@gmail.com (S.P.); mcbane.robert@mayo.edu (R.M.); 5 Department of Cardiology, Mayo Clinic, Rochester, MN 55905, USA; wysokinski.waldemar@mayo.edu; 6 Midwestern University, Glendale, AZ 85308, USA; heidi.esser.85@gmail.com

**Keywords:** anticoagulants, serum levels, reversal agents

## Abstract

Millions of patients in the United States use anticoagulation for a variety of indications, such as the prevention of stroke in those with atrial fibrillation (AF) and the treatment and prevention of venous thrombosis. For over six decades warfarin was the only available oral anticoagulant, but now several DOACs are available and their use has become more prevalent in recent years. In spite of this increased use, many physicians remain reluctant to prescribe DOACs due to concerns about bleeding and reversibility.

## 1. Introduction

A 79-year-old woman experiencing an episode of atrial fibrillation and rapid ventricular rate was admitted to the hospital, where she was successfully cardioverted and started on apixaban and metoprolol. A week after discharge home, she accidently tripped and fell onto the edge of the dining room table and fractured her hip. The surgical team was concerned about operating emergently because the patient was prescribed apixaban, an anticoagulant that increases her risk of bleeding. There are no Food and Drug Administration (FDA) approved laboratory assays to monitor serum drug levels or a reversal agent available if the patient were to experience significant bleeding during surgery.

Though the patient eventually had a successful operation, she had to endure three extra hospital days of waiting, bedridden and heavily medicated. Such delays in orthopedic surgery have been described as increasing the risk of both minor and major medical complications [1,2,3]. This case highlights a clear need for laboratory monitoring and reversal agents for direct oral anticoagulants (DOACs), the nomenclature recommended by the society of Thrombosis and Haemostasis for medications formerly known as Novel Oral Anticoagulants (NOACs). The purpose of this review is to discuss the clinical application, serum level measuring and reversal agents of DOACs.

Millions of patients in the United States use anticoagulation for a variety of indications, such as the prevention of stroke in those with atrial fibrillation (AF) and the treatment and prevention of venous thrombosis. DOACs seems to have better safety profile with almost ½ the risk of intracranial bleeding. For over six decades warfarin was the only available oral anticoagulant, but now several DOACs are available and their use has become more prevalent in recent years [4]. In spite of this increased use, many physicians remain reluctant to prescribe DOACs due to concerns about bleeding and reversibility [5].

Warfarin is a vitamin K antagonist discovered in the 1920s after cows that had eaten moldy sweet clover died of hemorrhage following minor trauma. Since the Wisconsin Alumni Research Foundation (WARF) funded part of the work, the isolated compound became known as warfarin. For a long time, warfarin was primarily used as rat poison [6]. However, due to its favorable efficacy as an anticoagulant and cost profile [7,8,9,10,11] over time warfarin became more popular in the clinical setting, and it is now one of the World Health Organization’s Essential Medicines [12] and the most commonly used anticoagulant worldwide. Nonetheless it is also the number one medicine to cause emergency room visits and complications [13]. Warfarin is easy to reverse and has well-established reversal protocols (using prothrombin complex), although these may differ across different countries (see Table 1 for U.S. guidelines for warfarin reversal) [14]. There are several well-known problems with warfarin, including frequent International Normalized Ratio (INR) monitoring, labile INR in many studies [15,16,17,18], numerous drug and food interactions [19], and a risk of bleeding [20,21,22]. Concern over these issues can cause providers to underuse warfarin, perhaps increasing morbidity and mortality [19,23,24,25,26,27].

In 2010, the Food and Drug Administration (FDA) approved its first DOAC, dabigatran (Pradaxa, Boehringer Ingelheim), followed by rivaroxaban (Xarelto, Johnson and Johnson and Bayer Healthcare AG) in 2011, apixaban (Eliquis, Bristol-Meyers Squibb and Pfizer Inc.) in 2012 and edoxaban (Savaysa/Lixiana, Daiichi Sankyo) in 2015 [28]. The doses used for stroke as well as Venous thromboembolism (VTE) and pulmonary embolism (PE) are shown in Table 2. Several other DOACs previously in development, such as darexaban (Astellas Pharma), eribaxaban (Pfizer Inc.) and letaxaban (Takeda) [29], were discontinued by their drug companies after Phase II clinical trials. Betrixaban (Portola Pharmaceuticals) was tested in Phase II trials for VTE prophylaxis post total-knee replacement and stroke prevention in AF [30,31]; neither trial showed significant difference in efficacy when compared to enoxaparin (for VTE) or warfarin (for stroke), respectively. This DOAC was also trialed in medically ill patients for VTE prevention, and compared to enoxaparin, betrixaban was shown to have benefit; however, it was not clinically significant (*p* = 0.054, 95% confidence interval 0.65–1.0 [32].

As of 2016, DOAC prescriptions exceeded those for warfarin in outpatient office visits for atrial fibrillation, with rivaroxaban being the most frequently prescribed DOAC (47.9%), followed by apixaban (26.5%), and dabigatran (25.5%) [33]. This equated to 4,210,000 prescriptions for DOACs in the US.

Routine coagulation assays, including prothrombin time (PT) and activated partial thromboplastin time (aPTT), cannot be used to reliably monitor therapeutic anticoagulation with DOACs [34,35,36]. There are no FDA approved methods or reagents to monitor DOACs, but specific tests for these anticoagulants are available only in a few US laboratories.

Unlike the widely available interventions for reversing warfarin anticoagulation (e.g., vitamin K supplementation or prothrombin complex concentrates), there is currently only one FDA approved reversal agent for the DOAC class of anticoagulants. Traditional methods for reversing anticoagulation or managing bleeding patients in the setting of anticoagulation have not shown consistent results.

## 2. Direct Thrombin Inhibitors

### 2.1. Dabigatran

#### 2.1.1. Clinical Application

Dabigatran is approved by the FDA for stroke prevention in non-valvular (NV) AF and for prophylaxis and treatment of VTE. This drug has been evaluated at doses of 110 and 150 mg and is currently approved at 150 mg twice daily for those with a creatinine clearance (CrCl) of >30 mg/dL and at 75 mg twice daily for those with a CrCl of 15–30 mg/dL [37].

The Randomized Evaluation of Long-Term Anticoagulation Therapy (RE-LY) trial of dabigatran in non-valvular atrial fibrillation (NVAF) patients compared dabigatran to warfarin [15]. Dabigatran at a dose of 150 mg twice daily was associated with lower rates of both stroke (including intracranial hemorrhage) and systemic embolism [15,38]. Although in both RE-LY [15] and another study by Graham et al [38] it was associated with higher rates of gastrointestinal (GI) hemorrhage in men >85 years and women >75 years of age, the 110 mg dose demonstrated similar efficacy with less bleeding than warfarin and no increase in (GI) bleeding vs warfarin. It was reported that among those who did experience hemorrhage on either dabigatran or warfarin, the dabigatran cohort experienced shorter ICU stays [38]. The Twice-daily Oral Direct Thrombin Inhibitor Dabigatran Etexilate in the Long-Term Prevention of Recurrent Symptomatic VTE (RE-SONATE) and Secondary Prevention of Venous Thrombo Embolism (VTE) (RE-MEDY) trials [39] compared dabigatran versus warfarin treatment of long-term VTE treatment, finding that dabigatran was significantly associated with lower rates of VTE although it had more major and clinically relevant bleeds.

The RE-COVER trial [40] compared dabigatran versus warfarin for rates of recurrent VTE in patients who had already experienced a VTE. It concluded that dabigatran was non-inferior for preventing recurrent VTE and that there was no difference in major bleeding. The study also found that dabigatran was associated with less major and clinically relevant non-major bleeds.

The RE-NOVATE [41] trial examined the difference in VTE post-total hip replacement (THR) between dabigatran 220 mg and 150 mg and enoxaparin. It was found that dabigatran in both doses was non-inferior to enoxaparin in regard to VTE prophylaxis and there was no difference in major bleeding.

Finally, the RE-MOBILIZE [42] trial looked at dabigatran versus enoxaparin 30 mg BID for VTE prophylaxis after knee replacement, finding that dabigatran had inferior efficacy to enoxaparin and that both had similar bleeding rates.

Dabigatran is a powerful anticoagulant with similar risk of bleeding and increased risk of gastrointestinal bleeding as compared to warfarin, but it should be avoided in elderly patients with renal impairment and those with GI pathology.

#### 2.1.2. Laboratory Monitoring of Dabigatran 

Mass spectrometry is the gold standard for measuring DOAC concentrations, but these assays are not routinely available in most laboratories [43]. Dabigatran levels can also be measured using dilute thrombin time, aPTT and ecarin clotting time (ECT) [44,45,46,47,48]. However, like mass spectrometry, few laboratories in the USA offer these tests. The standard, widely available thrombin time is too sensitive to measure dabigatran levels; however, a normal thrombin time does indicate that there is minimal to no dabigatran present in the blood sample [47,49].

#### 2.1.3. Reversal of Dabigatran

Fresh frozen plasma (FFP), PCC, activated prothrombin complex concentrates (aPCC), and recombinant factor VIIa (rFVIIa) have been tested for reversing the effects of dabigatran [46,50,51,52,53]. FFP contains all the clotting factors and is traditionally dosed at 10–15 mL/Kg to reverse coagulopathy. Large volumes are often required to control bleeding, and because FFP is stored in a frozen state, there may be critical delays in emergencies [54]. PCC contains inactivated clotting factors II, IX, and X and a variable amount of factor VII (hence either three-factor or four-factor). Some PCC also contains the natural anticoagulant factors proteins C and protein S to help mitigate the risk of thrombosis and limit activation of factor concentrate. PCC is stored as lyophilized powder and is quickly reconstituted with diluent allowing for rapid availability in critical situations [55]. aPCC contains the activated rFVII and inactivated factors II, IX, and X. The activated complex is approved to prevent or control bleeding patients with congenital or acquired hemophilia who have factor VIII inhibitors [56]. rFVIIa activates the extrinsic pathway and therefore increases fibrin formation and decreases INR. It is used off-label for warfarin reversal, trauma and surgery-induced coagulopathy, cardiac surgery, and intracranial hemorrhage. Like many of the other agents, it increases the risk of thrombosis [57,58].

Many studies have investigated these various potential reversal options. Two animal studies [50,51] showed that PCC reversed dabigatran-induced hematoma expansion and bleeding in a dose-dependent manner, although another animal study found it ineffective for reversing aPTT, thrombin time, dilute thrombin time, or ECT [51]. The off-label use of PCC should be considered in first line of treatment. FFP was studied in murine models, where it decreased the size of induced intracranial hemorrhage in high but not low-dose dabigatran and had no effect on overall mortality [50]. FFP successfully decreased bleeding time in induced rat tail incision; however, thrombin time, aPTT, and ECT were not improved [50].

In 2015, a specific reversal agent (antidote), idarucizumab (Praxbind, Boehringer Ingelheim Pharmaceuticals), a monoclonal antibody fragment that binds specifically to dabigatran, was introduced and approved by the FDA. Idarucizumab immediately and completely reverses the effects of dabigatran in a dose-dependent manner, as measured by dilute thrombin time and ecarin clotting time [59,60,61,62,63]. The REVERSE-AD [62] trial studied idarucizumab in emergency situations such as major bleeding and in those requiring emergent surgery and has shown effective and rapid reversal without any identified safety concerns. Full results in 2017 showed that 100% of patients with life threatening bleeding had reversal of diluted thrombin time and ecarin clotting time and 67.7% had cessation of bleeding within 24 hours; in those who needed reversal for urgent surgery, 93% had had cessation of bleeding within 24 hours. Mortality was 13.5% in life-threatening bleeding group and 12.6% in the urgent surgery group and thrombosis was 4.8%; there was no control group [63]. Hopefully, there will be more trials or retrospective data reviews to confirm safety in this target population. Adverse events to idarucizumab thus far have been rare and mild, such as injection site erythema or bruising, and the agent was found to be more efficacious than (PCC) in porcine and human models [64,65,66]. Idarucizumab is currently approved by the FDA for use in those requiring surgery or procedures that cannot be delayed and in those with life-threatening or serious bleeding. Death and thromboembolic events have been reported at 30 days, however, could be related to comorbidities.

Another reversal agent currently in Phase II trials is PER977 or ciraparantag (aripazine, Perosphere), which binds directly to anticoagulants and inactivates them based on animal models. It was found to reverse dabigatran, along with unfractionated heparin, low molecular weight heparins, and factor Xa inhibitors [67,68,69,70,71]. Studies of ciraparantag are ongoing; however, it appears to be safe and well-tolerated [72].

### 2.2. Bivalirudin

#### 2.2.1. Clinical Application

Bivalirudin is a parenteral reversible thrombin inhibitor and has a very short half-life of 25 min. It can be used for anticoagulation in heparin-induced thrombocytopenia (HIT) [73,74] and can also be used in percutaneous coronary intervention (PCI) and percutaneous transluminal coronary angioplasty (PTCA) [74,75,76,77].

#### 2.2.2. Laboratory Monitoring of Bivalirudin

Since it is partially eliminated by the kidneys, the dosing of bivalirudin must be adjusted for those with renal impairment. For those with no hepatic or renal impairment, it is typically dosed IV at 0.15 mg/kg/h to target aPTT values 1.5–2.5 times the baseline value [78]. It is not typically used for stroke/systemic embolism prophylaxis, or DVT/PE treatment/prophylaxis since it is not available orally [72]. Bivalirudin levels are often measured with ECT or PTT [79,80]. Zucker et al. suggested that a modified activated clotting time (ACT) may be an option after noting that the ACT had a good correlation with bivalirudin levels used in cardiopulmonary bypass [80].

#### 2.2.3. Reversal of Bivalirudin

Bivalirudin has no approved reversal agent. A study was conducted on the blood of eight patients who had been on Bivalirudin, ex-vivo, and found that rFVIIa significantly (*p* < 0.01) decreased aPTT compared to placebo and also similarly reversed anticoagulation as measured by thromboelastography (TEG) [81,82]. Another study explored whether bivalirudin could be reversed by idarucizumab. However, idarucizumab is highly specific for dabigatran, and other drug classes like factor-Xa inhibitors did not demonstrate reversal as measured by anti-factor Xa activity, nor did agents like bivalirudin or argatroban [83].

### 2.3. Argatroban

#### 2.3.1. Clinical Application

Argatroban is a parenteral direct thrombin inhibitor that, like bivalirudin, is approved for use in heparin-induced thrombocytopenia. It has a half-life of 24 min and is given IV adjusted to a target aPTT of 1.5–3 times the baseline as long as that number is less than 100 seconds. Unfortunately, it can lead to falsely elevated INR, which complicates the transition to warfarin. Argatroban can also be used in percutaneous coronary intervention in patients who are at a high risk of HIT. Since this drug is hepatically metabolized, it is best used for HIT patients with renal impairment. It must be dose-adjusted for those with hepatic impairment (although fondaparinux or Bivalirudin would be preferred in this setting) [84].

#### 2.3.2. Reversal of Argatroban

The anticoagulant effect of Argatroban is reversed with rFVIIa as measured by TEG [82]. It was not reversed by ciraparantag, a novel agent that is under evaluation as a reversal for factor Xa inhibitors and dabigatran [85].

## 3. Factor Xa Inhibitors

Factor Xa inhibitors include rivaroxaban (Xarelto), apixaban (Eliquis), edoxaban (Savaysa), betrixaban (Bevyxxa).

### 3.1. Drug Overview

#### 3.1.1. Rivaroxaban

Rivaroxaban is a factor Xa inhibitor that is dosed once daily from 10–20 mg depending on indication [86,87] (Table 2). Trials regarding the use of rivaroxaban in atrial fibrillation have found it to be non-inferior to warfarin in preventing stroke or systemic embolism [88,89,90], even in patients with prior stroke or transient ischemic attack [91]. Bleeding risk was largely similar between warfarin and rivaroxaban; however, the ROCKET-AF [88] study found that bleeding was less likely to be fatal with rivaroxaban and that there was less intracranial hemorrhage but more GI hemorrhage in patients taking rivaroxaban. The recommended dosing is 20 mg once daily for the prevention of stroke and systemic emboli and 15 mg daily in patients with CrCl 15–50 mL/min.

For the indication of VTE/PE (dosed at 15 mg twice daily for 3 weeks followed by 20 mg daily thereafter) rivaroxaban was non-inferior compared with enoxaparin and warfarin, in two studies [92,93]. Patients on rivaroxaban had less bleeding than enoxaparin in the PE trial [16]. In studies comparing rivaroxaban and enoxaparin in prevention of VTE after orthopedic surgery, rivaroxaban was found to be non-inferior in three and superior in one, with no differences in bleeding [92,93,94,95]. The rivaroxaban for thromboprophylaxis in acutely ill medical patients (MAGELLAN) trial the ten-day cohort, each group had a 2.7% odd of VTE, and rivaroxaban was non-inferior. In the longer 35-day cohort, rivaroxaban had less VTE (2.7 versus 5.7%), meeting the criteria for superiority. Rivaroxaban, found to be non-inferior to low molecular weight heparin, however, was associated with an increased risk of clinically relevant bleeding [96,97].

Rivaroxaban has also been evaluated for use in acute coronary syndrome (ACS) [98]. In ACS patients, rivaroxaban 2.5 mg reduced a composite endpoint of death from cardiovascular causes, MI, or stroke compared to placebo. It also reduced all-cause mortality and stent thrombosis compared to placebo. However, it was also found to significantly increase the rate of major bleeding unrelated to coronary artery bypass graft versus placebo, minor bleeding, and intracranial hemorrhage versus placebo [98].

Rivaroxaban was recently trialed in patients with arterial disease versus antiplatelet agents. Rivaroxaban and aspirin combined showed a lower rate of cardiovascular death, myocardial infarction (MI), or stroke whereas rivaroxaban alone versus aspirin showed no difference in endpoint but reduced rates of amputation. There was more bleeding in the group with rivaroxaban plus aspirin and rivaroxaban alone versus aspirin [99].

Rivaroxaban has interactions with several different medications, some of which are considerable. For example, many cytochrome P450 inducers and inhibitors can influence its anticoagulant effect [100].

#### 3.1.2. Apixaban

Apixaban is a factor Xa inhibitor that is dosed at 2.5 mg twice daily for VTE prophylaxis and 5 mg daily for stroke prophylaxis in NVAF. The dosing is halved if patients are on inhibitors of P450 3A4 such as ketoconazole, itraconazole, ritonavir, or clarithromycin. It is also halved if two or more of the following apply: age greater than 80 years, weight less than or equal to 60 kg, and creatinine greater than or equal to 1.5 mg/dL [101]. Other dosing alterations include those made for persons who cannot take pills or for pediatric patients [34]. One study indicates there is similar bioavailability between crushed and oral apixaban [102]. Another study is investigating apixaban in the pediatric population at risk for thrombosis [103].

Numerous clinical trials have been performed to evaluate the efficacy of apixaban. For example, in atrial fibrillation patients, apixaban was found to be superior in stroke and systemic embolism prevention when compared to aspirin or warfarin. Bleeding rates were also in favor of apixaban [104,105]. In regard to treatment for VTE, apixaban was shown to be non-inferior to enoxaparin [106,107]. For VTE prophylaxis post-orthopedic surgery, apixaban was superior to enoxaparin in two studies [108,109] but did not meet criteria for non-inferiority in another [110]. Two of these studies showed less bleeding with apixaban and the other showed increased bleeding with apixaban as compared to enoxaparin. In a trial of VTE prophylaxis in the medically ill, apixaban did not meet the criteria for superiority versus enoxaparin and had more bleeding [17].

#### 3.1.3. Edoxaban

Edoxaban is a once-daily pill that comes in doses of 30 and 60 mg that was approved by the FDA in January 2015 for use in stroke prevention in atrial fibrillation and for prophylaxis and treatment of VTE. It is prescribed at 60 mg doses to prevent stroke or systemic embolism and for PE/DVT treatment, or at 30 mg doses for DVT treatment if the patient weighs <60 kg. ENGAGE-TIMI AF 48, the main trial in atrial fibrillation, showed that edoxaban was superior to warfarin for prevention of stroke and had lower rates of major bleeding, death, and hemorrhagic stroke, although it had higher rates of GI bleeds in the 60 mg group and lower rates of bleeding in the 30 mg dose group [111]. In a Phase II trial there was increased bleeding with higher doses of edoxaban as compared to warfarin [112]. For use in treating VTE, edoxaban was non-inferior to warfarin for VTE recurrence prevention and superior in regard to clinically relevant bleeding [113].

### 3.2. Laboratory Monitoring of Direct Factor Xa Inhibitors

There are no commercial, FDA-approved assays for direct factor Xa inhibitor measurements in the United States. Modified chromogenic anti-Xa assays that have been calibrated with the specific drug (rivaroxaban, apixaban or edoxaban) are not widely available. A normal PT levels indicate complete clearance of the Xa inhibitor. Both TEG and thrombin generation assay (TG) are increased in the presence of a Xa inhibitor. Anti-Xa assays calibrated with unfractionated heparin (UFH) and/or low molecular weight heparin (LMWH) cannot be used to measured direct factor Xa inhibitor concentrations for several reasons, including that the cut off levels (30, 50, and 100 ng/mL) have not been clinically validated. Additionally, different units of measure (IU/mL vs. ng/mL) and therapeutic ranges for the direct factor Xa inhibitors that far exceed the calibration range of the UFH/LMWH anti-Xa assay [114,115].

### 3.3. Reversal of Direct Factor Xa Inhibitors

There are no approved reversal agents for factor Xa inhibitors, but several potential candidates have been assessed. The short half life of the DOACs compared to warfarin enable faster clearance of the drug in patients with normal renal function. An in vitro study evaluated PCC, activated PCC, and rFVII for rivaroxaban-induced changes in prothrombin time (PT), TG and in thromboelastometry clotting time (CT) in whole blood in healthy subjects [116]. All three were only 50% effective in reversing PT, TG, and CT. Another study indicated that PCC was not effective in reversing changes to aPTT, PT ratio, or PT levels [116]. Levi et al. compared three versus four-factor PCC effects on rivaroxaban-induced changes in PT and TG. It was found that four-factor worked better for reversing PT and three-factor worked better for thrombin generation. It was not clear whether this was simply due to differences in pro-coagulants among the patients or whether this was a legitimate finding [117]. However, an in vivo study of 12 healthy male subjects showed that rivaroxaban but not dabigatran can be completely and immediately reversed by PCC [118]. More studies need to be performed with larger sample sizes that also include women.

A more recent trial looked at reversal of apixaban and rivaroxaban in emergency bleeding events with PCC and found that 70.2% of patients had reversal while 30.9% did not; 32% died within 30 days, and overall the patients had low VTE rate [119].

Andexanet alfa, a Factor Xa reversal agent, is undergoing evaluation with promising initial studies. Andexanet alfa is an investigational drug to reverse factor Xa inhibitors such as rivaroxaban, apixaban, edoxaban, and the investigational drug betrixaban. It acts as a factor Xa decoy protein that affects both direct and indirect Xa inhibitors. The ANNEXA-R trial [120] specifically investigated the reversal of rivaroxaban in healthy volunteers and showed that there was a 92% reduction of anti-factor Xa activity compared to baseline, which was highly significant compared to placebo. There were no serious or severe adverse events reported, including no thrombosis. Thrombin generation returned to pre-rivaroxaban levels. In the ANNEXA-A trial [120], andexanet was found to be an effective reversal agent for apixaban. There were no major adverse effects reported, including no evidence of increased thrombosis. Thrombin generation was restored to pre-apixaban levels within 2–5 min with administration of andexanet.

Currently, the ANNEXA-4 trial (on-going) is in Phase 3B/4 and is examining the efficacy of Andexanet in reversing the anticoagulation effect of rivaroxaban, apixaban, and edoxaban. Portola Pharmaceuticals, the ANNEXA-4 sponsor, has released the first set of results on reversal of rivaroxaban and apixaban-induced anti-factor Xa activity and acute major bleeding. After administration of andexanet, anti-factor Xa levels decreased by a mean of 89% in those on rivaroxaban and 93% for those on apixaban. After 12 h, 79% in both groups were deemed to have good or excellent clinical hemostasis. In the next 30 days, 18% had thrombotic events. The trial’s subjects were older and more ill than those who do not require reversal of anticoagulants and thus more prone to thrombosis (including stroke, myocardial infarction, and DVT) and heart failure [121].

Andexanet is considered to be a FDA-designated breakthrough therapy, and a Biologics License Application was submitted to the FDA in February 2016, in August, the FDA asked for more information on manufacturing and clinical data. Portola Pharmaceuticals is addressing these requests and a decision on approval is expected in February 2018.

Another therapy previously mentioned, PER77 or ciraparantag, was examined in Phase II clinical trials in attempts to reverse multiple anticoagulants and was found to reverse unfractionated heparin, enoxaparin, and DOACs including edoxaban and dabigatran [122] (Table 3).

## 4. Conclusions

In summary, the DOACs are a promising group of drugs that have been shown to be non-inferior to warfarin and generally have fewer bleeding risks and less fatal bleeding. However, there are no widely available laboratory assays to measure drug levels in patients, and there are concerns amongst physicians about reversing these agents in the event of bleeding or in a patient requiring emergency surgery. Indeed, there are no current guidelines about emergency reversal of these agents, but reversal agents are being developed. Idarucizumab was recently approved by the FDA, andexanet is in the final stages of FDA approval, and ciraparantag is currently in clinical trials.

## Figures and Tables

**Table 1:** Warfarin Reversal: American College of Chest Physicians Guidelines.

INR	No Bleeding	Requires Urgent Reversal	Life Threatening Bleeding
<4.5	Hold warfarin until INR in range	Hold warfarin	Hold warfarin
			Vitamin K 10 mg IV over 30 min
		Consider Vitamin K 2.5 mg PO	
			4U FFP or Kcentra [1]
4.5–10	Hold warfarin	Hold warfarin	Hold warfarin
			Vitamin K 10 mg IV over 30 min
	Consider 2.5 mg vitamin K PO	Vitamin K 2.5 mg PO or 1 mg IV	
			4U FFP or Kcentra [2]
>10	Hold warfarin	Hold warfarin	Hold warfarin
			Vitamin K 10 mg IV over 30 min
	Vitamin K 2.5 mg PO or 1–2 mg IV over 30 min. Repeat Vitamin K every 24 h as required	Vitamin K 1–2 mg IV over 30 min. Repeat every 6–24 h as required	
			4U FFP or Kcentra [3]

Note: 1. If INR 1.5–3.9, use 25 units/kg, maximum 2500 units; 2. If INR 4–6, use 35 units/kg, maximum 3500 units; 3. If INR > 6, use 50 units/kg, maximum 5000 units. FFR: fresh frozen plasma; INR: International normalized ratio; IV: intravenously; PO: per os (by mouth); U: unit.

**Table 2:** Summary of DOACs doses used in stroke/systemic embolism prophylaxis and DVT/PE treatment.

DOAC	Mechanism	Typical Doses (mg)	Renal Elimination (%)	Half-Life (h)
Dabigatran	Direct thrombin inhibitor	110, 150, 75	80	12–14
Apixaban	Factor Xa inhibitor	2.5, 5	25	8–15
Rivaroxaban	Factor Xa inhibitor	10, 15, 20	33	9–13
Edoxaban	Factor Xa inhibitor	30, 60	35	8–10

**Table 3:** Reversal Guide.

Anticoagulant	FDA Approved-Specific Reversal Agent	Other Reversal Agents			
		rFVIIa	4 Factor PCC (Kcentra)	Andexanet (Pending FDA Approval)	Ciraparantag (Not Tested in Human)
Unfractionated Heparin	Protamine. 1 mg/100 units heparin	Unknown	-	Yes	Yes
LMWH	Protamine. 1 mg/L mg enoxaparin or 1 mg/100 units anti-Xa (dalteparin, tinzeparin)	Unknown	-	Yes	Yes
Dabigatran	Idarucizumab 5g dose (dose can be repeated)	No	Some studies	No	Yes
Xa inhibitors	None	Partial	Likely	Yes	Yes
